# A novel bat pollination system involving obligate flower corolla removal has implications for global *Dillenia* conservation

**DOI:** 10.1371/journal.pone.0262985

**Published:** 2022-02-03

**Authors:** Sophie Petit, Annette T. Scanlon, Alivereti Naikatini, Tara Pukala, Russell Schumann

**Affiliations:** 1 UniSA STEM, University of South Australia, Mawson Lakes, South Australia, Australia; 2 NatureFiji-MareqetiViti, Suva, Fiji Islands; 3 Kangaroo Island Research Station, Dudley West, South Australia, Australia; 4 South Pacific Regional Herbarium and Biodiversity Centre, Institute of Applied Sciences, University of the South Pacific, Private Bag, Laucala Campus, Suva, Fiji Islands; 5 School of Physical Sciences, University of Adelaide, Adelaide, South Australia, Australia; Indian Institute of Science, INDIA

## Abstract

The Dilleniaceae is known to produce nectarless flowers pollinated by bees, but the fact that bats ingest *Dillenia biflora* pollen led us to question pollination assumptions for these trees. We aimed to identify the pollinators of *D*. *biflora*, check for nectar presence, and investigate potential for cleistogamy and global prevalence of this pollination system. We examined aspects of the pollination of *D*. *biflora* on two Fijian islands using video recordings, direct observations, hand pollination, measurements (flowers, bite marks, nectar), and monitoring. The flowers, receptive for one night, contained copious nectar and had permanently closed globose corollas that required removal by bats for pollination. All the 101 flowers that retained their corolla died and did not produce seeds by cleistogamy. The bat *Notopteris macdonaldi* was well adapted to corolla removal. Keeping corollas closed until bats manipulate the nectar-rich flowers is a beneficial strategy in high-rainfall environments with many flower parasites. We propose to name a pollination system reliant exclusively on bats “chiropteropisteusis.” From clues in the literature, other species in the geographical range of *Dillenia* are probably chiropteropisunous. Chiropteropisteusis should be investigated in the Old-World range of *Dillenia*, many species of which are threatened. The remarkable “fall” of the entire corolla observed by an earlier botanist for several species in the genus is most likely attributable to bats. This discovery has important implications for the conservation of bat-dependent trees and their associated fauna, particularly considering the high level of threat faced by flying-foxes globally.

## Introduction

Chiropterophilous plants attract bat visitors with specialized traits such as bell-shaped flowers that facilitate acoustic detection [1], scent [2], UV reflectance [3], and abundant floral nectar [4]. Willmer [5], in agreement with Faegri and van der Pijl’s [6] well-known syndrome of chiropterophily, lists a series of flower characteristics that prevail in this syndrome: nocturnal anthesis and nectar secretion, flowers lasting one night on plants with long flowering times, large container-shaped flowers (some brushes), flowers away from leaves, thick corolla wall, pale colours, strong scents, numerous stamens rich in pollen, large nectar volumes, large space between nectar and stigmas and anthers.

Known to produce nectarless flowers [7, 8], the family Dilleniaceae with ~ 500 mostly pantropical species [9] contains the genus *Dillenia*, whose large and stout flowers are assumed to be pollinated principally by bees [10], yet have many of the characteristics highlighted by Willmer for bat flowers [5]. Dilleniaceae is of particular interest to evolutionary biologists because its phylogenetic placement “*remains one of the last major mysteries in the angiosperms*” [8]. Of 22 *Dillenia* species assessed by the IUCN, 17 are threatened. The genus is distributed from eastern Madagascar to Fiji and from the southern Himalayas, southwest China and Hainan to the northeast coast of Queensland in Australia [7, 11], but the pollinators of most species are unknown, including for the important coloniser tree *D*. *biflora*, which occurs in Fiji’s and Vanuatu’s rainforests. Conservation International [12] chose *D*. *biflora* as one of the key native species to use in reforestation in northern Viti Levu, Fiji. In this country, it is used for timber and medicine, and is also habitat for endangered frog species [13]. Its fruits and seeds are likely consumed by both birds and bats.

*Dillenia biflora* pollen was recorded in 70% of the dietary samples of the Vulnerable cave-dwelling blossom bat *Notopteris macdonaldi* on Vanua Levu [13], leading us to presume that this flying-fox was involved in the pollination of *D*. *biflora*. The common *Pteropus tonganus* also consumed *D*. *biflora* pollen (15% of diet samples), as did *Pteropus samoensis* (12% of samples [13]). We observed that the petals of *D*. *biflora* flowers did not open, forming a globose corolla acting as a lid over the anthers and multilobed stigma. The presence of pollen in the bats’ diet indicated that the flowers may not be cleistogamous (cleistogamy = self-pollinated, permanently closed flowers), and we hypothesized that they were pollinated by bats.

In view of the ecosystem role of *D*. *biflora* in reforestation, as a coloniser, and as habitat and food source for threatened vertebrates, it is very important to understand the reproductive mechanisms of this tree. The involvement of bats in its pollination should elicit immediate conservation concerns, since island bats are faced with numerous threats [14], including hunting and cave disturbance in Fiji [15]. In addition, demonstrating dependence on bats for a *Dillenia* tree species could indicate that other species in the wide geographic range of this genus are similarly dependent, and linked to the fate of local bats. The status of bats is poorly known in many developing countries. Understanding their role in the pollination of *Dillenia* as well as their dietary dependence on trees of this genus is critical for the management of agroforestry and conservation reserves where the taxa co-occur. Consequently, we investigated aspects of the pollination of *D*. *biflora* trees at Waisali on Vanua Levu and at Colo-i-Suva Forest Park on Viti Levu. We aimed to identify the pollinators of *D*. *biflora*, investigate potential cleistogamy, and determine whether flowers offered a nectar reward, contrary to what is known for the family. We also examined the literature for evidence of the global prevalence of this pollination system.

## Study sites and methods

### Study sites

We examined the pollination of 39 *D*. *biflora* trees at Waisali, Vanua Levu (16°38’42” S, 198°14’23” E), monthly from August 2009 to March 2011, and 19 trees at Colo-i-Suva, Viti Levu (18°03’44” S, 178°27’50” E), in November–December 2016 (summer in the southern hemisphere, also “wet season”) and an additional 10 (total 29) in June−July 2017 (winter, “dry season”). The Waisali site was located within the southern Drawa Catchment of the main mountain range of Vanua Levu; rainfall is 3000–6000 mm per year [16]. Relative humidity ranged from 93–98% and daily temperature from 19.2–34.6°C from September 2010–March 2011 (Hygrochron, iButton, Maxim Integrated, San José, U.S.A.). The catchment includes ~ 6000 ha of lowland and upland rainforest, which represents important remnant rainforest in Fiji [17] and encompasses Waisali Rainforest Reserve. The Colo-i-Suva Forest Park site followed the track bisecting another remnant forest, 11 km north of Suva, in the catchment for Nausori and Nasinu. The lowland Forest Park was logged and planted with mahogany trees in the 1940s and 1950s [18], stands of which remain among native vegetation regrowth. Dilleniaceae, consisting of *Dillenia biflora* and *Hibbertia luccens*, represented the 20^th^ most abundant family by number of individuals in a 6000-m^2^ plot at the adjacent Vago Forest Reserve, and all 81 individuals in that plot were *D*. *biflora* [19]. Site access was granted by the National Trust of Fiji (Waisali Rainforest Reserve), the community of Waisali (Waisali) and the Ministry of Forestry (Colo-i-Suva Forest Park). All necessary permits were obtained for the described study, which complied with all relevant regulations. Weather information for the area is in Text A in S1 Appendix.

### Study species

*Dillenia biflora* is in the clade Dilleniales in the eudicots. This class consists exclusively of the Dilleniaceae and its phylogenetic position in the angiosperms is unresolved [8]. A reported noteworthy character of the family is the absence of nectary [8]. The Plant List version 1.1 [20] shows 142 *Dillenia* species, with 58 accepted, 71 synonyms, and 13 unresolved. *Dillenia biflora*, known as kuluva by the local people, is a pioneer tree common in primary and secondary forests, as well as in open areas such as gardens [21]. It bears large, simple leaves, and is a steady-state flower producer [13], like others in the genus [10]. The large and sturdy (~ 3 cm calyx length) multi-staminate (200˗500 [21]) flowers with punctiform stigmas [7] are cream and white, sometimes yellow and pink, and borne singly and in pairs, or sometimes as triples in terminal leaf whorls. Most mature flowers were delicately perfumed, similarly to bat-pollinated *Stenocereus griseus* flowers in the Caribbean (SP, pers. obs., [22]); some had undetectable scents. Fruits are berries with translucent white fleshy funicular arils bearing small black seeds and enclosed by sepals during development. Sepals curve back when the fruit is mature, splitting when ripe [21]. Pollinators for *D*. *biflora* had not been recorded prior to our study, except anecdotally: a village elder (S. Tuicuvu, Waya Island 2008) told us that the flowers were visited by bats during summer.

At Waisali, *Notopteris macdonaldi*, a cave-dwelling Vulnerable bat [23] with peculiar long tail and dentition [24, 25] (adult mass = 51−89 g [26]), the Near Threatened *Pteropus samoensis* [27], and the Least Concern *P*. *tonganus* [28] all occurred [26]. These bats also existed on Viti Levu [15].

### Flower measurements and observations

**Fig 1:**
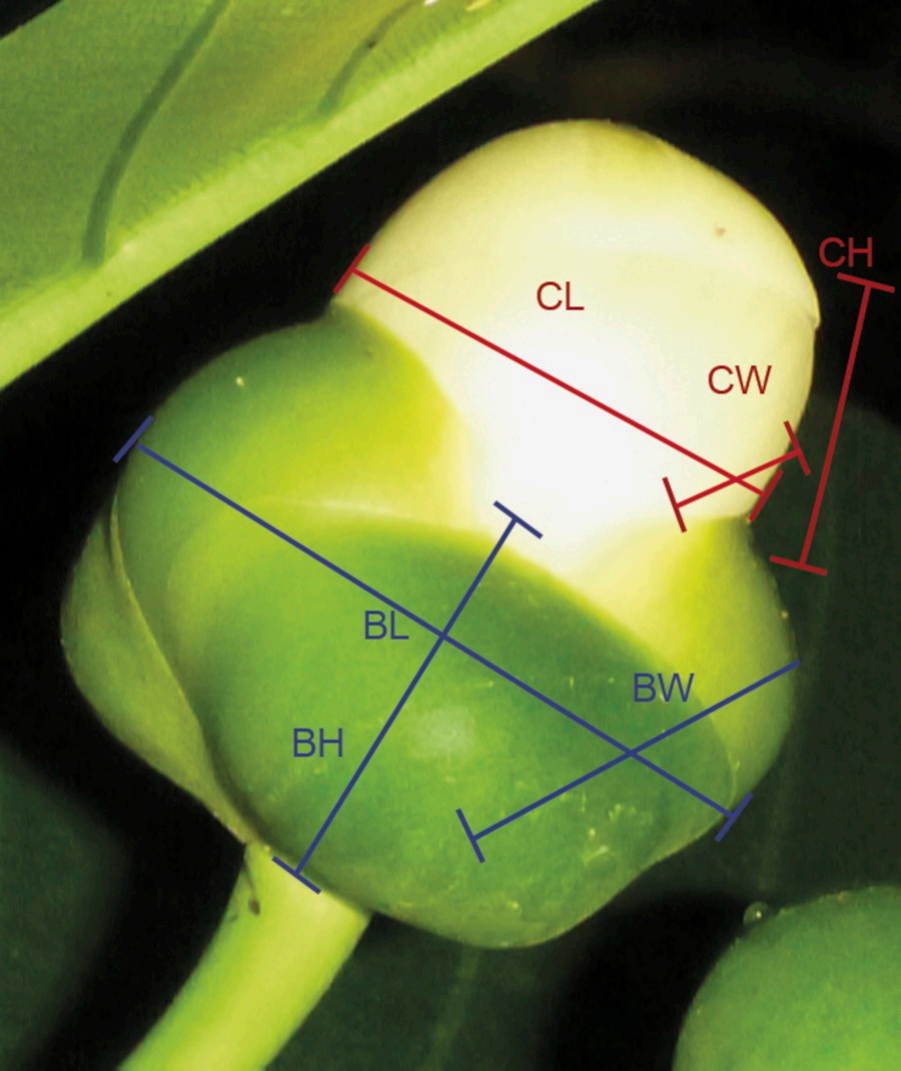
Measurements of mature flowers. Corolla height (CH), corolla width (CW), corolla length (CL), calyx height (BH), calyx width (BW), and calyx length (BL).

We measured 58 mature flowers (1˗34 per tree, n = 10 trees) at Colo-i-Suva using Vernier callipers. Measurements of flowers from the same tree were averaged, so each tree has n = 1 flower equivalent for the purpose of presenting mean measurements for the species. They comprised: corolla height, width, and length, calyx height, width, and length (Fig 1).

At Waisali, we studied the fate of *D*. *biflora* buds and flowers on a total of 29 trees. Some individual bud clusters (66 buds from 32 clusters on 19 trees in primary and secondary forests) were numbered and labelled using aluminium tags attached with copper wires (Forestry Tools, Willoughby, Australia), and their development followed to completion at least monthly. We inspected all leaf whorls on 10 trees in disturbed forest using a 12-m extension ladder and Mini Bullet CMOS camera mounted on a 12-m extendable pole with hand-held camcorder (Shenzhen Keyway Technology Development, Guangdong Province, China) monthly from August 2009 to March 2011 (except Jun. and Oct. 2010, n = 18 months). We also noted evidence of flower visitors during these surveys.

At Colo-i-Suva, Viti Levu, we worked with 29 *D*. *biflora* trees (4˗16 m high) located near the track of the Forest Park, so that in many cases we were able to erect a ladder on a utility truck in order to reach as many flowers as possible. In 2016 (summer), we marked mature flowers with tags made of synthetic white string and labelled (with permanent marker) flags of duct tape, but in 2017 (winter), we marked the flowers the day after they had become mature, in an effort to avoid disturbing bats and to maximize bat visitation. To increase sample size, we also monitored from the ground flowers that we could not reach, by using binoculars and mapping them.

In 2016, 1˗18 flowers were used per tree for 19 trees (median = 4.0; IQR = 8.0). The number of flowers in treatments depended on availability and priority, considering the difficulty of working in rainforest canopy at night and the relatively low number of flowers. We applied the following treatments to mature flowers: natural pollination (flowers with visitor removing the corolla, n = 25), no visitation (50 flowers had their corolla naturally left intact at maturity, 1 was bagged during the night after corolla removal by us, 2 had their corolla removed by us during the day, and 1 was bagged for 24 h; total n = 54), nocturnal cross-pollination by hand (with or without nectar taken for another study, n = 7), nocturnal self-pollination by hand (with or without nectar taken for another study; total n = 12), and diurnal self-pollination by hand (with or without nectar taken for another study; total n = 5). We also monitored flowers from which we had removed nectar during the day (n = 6), and during the night (n = 9). We hand-pollinated by wetting with distilled water the whole paper stem of a cotton applicator [29] and rubbing it against the anthers and stigmas of the same flowers (self-pollination) for ~ 20 s, or wrapped the paper stem of the cotton applicator loosely in aluminium foil before pollinating a different virgin flower with it. We bagged with black tulle bags held by strings all the flowers (calyxes) of which we had removed the corollas, and left the bags until the calyxes were tightly closed (~ 1 d). After our departure from Fiji, a Forestry Park Ranger monitored abortions and fruit maturity.

Few flowers were available in 2017, so although 21 trees were used, many of the flowers were from one heavily flowering tree near the Forestry station (tree 54), in an open area. We used 1 to 46 (the latter for tree 54) flowers per tree (median = 2.0; IQR = 2.0). Treatments were natural pollination (n = 42), no visitation (n = 18), nocturnal self-pollination (n = 31), and nocturnal cross-pollination (n = 4); we also monitored flowers from which we had collected nectar (n = 9; tree 54) (Table A in S1 Appendix). As previously, all the flowers from which we removed the corollas were bagged with black tulle. The Forestry Park Ranger was unavailable for two weeks before completing the fruit monitoring, so some closed calyxes (corollas removed) disappeared, and it is impossible to determine whether they aborted or became mature fruits. However, all intact flowers were followed to abortion. We do not have fruit collection dates for 2017, but rather age in number of days of the closed flower (potential fruit) on 10 August 2017.

### Flower visitors

At Colo-i-Suva in 2016, we used three Ultrafire and four Hyperfire cameras (Reconyx, Inc., Holmen, U.S.A.), but none of them triggered on flower visitors. So in 2017, we used four police body cameras with infra-red night vision, fixed wide-angle lens, and the ability to film continuously (model AS-PPR71; ASTR Industrial Co., Ltd., Shenzhen, China). We placed the cameras on a nearby tree (preferably within 3 m, but distance varied) soon after sunset (~ 1745 h) and started the continuous filming. We confirmed that a Reconyx camera did not trigger when a bat visited a flower, because the bat was recorded by the ASTR camera during a 38-s approach to flower 7.1 (2016), for which both camera types had been set. We observed flowers and their visitors over 224 h of video. We calculated approach time as time when the bat landed on the leaf whorl presenting the flower minus time when the bat was first heard or seen on the video. Handling time was time of corolla removal minus time of landing, and feeding time was time at which the bat stopped feeding minus time at which it started to feed (= time of corolla removal for first visit). We also observed different species of moths and geckoes at flowers. It was not possible to determine numbers and identify all species with certainty, because some were witnessed as eye shines, photographs were not detailed enough, or several animals may have been interested in the same flowers. However, we were able to monitor the behaviours of some of these animals at flowers for several minutes to several hours (e.g., two geckoes fed and fought at flower 46.2 for over 4 h; some animals were at the flower before we arrived). The research was approved by the UniSA Animal Ethics Committee (permits U35-16 and U10-17).

We examined the teeth and measured the gaps between the canines of the two *N*. *macdonaldi* specimens lodged at the South Pacific Herbarium (AXM 001 adult female and AXN 001 juvenile male, accession number SUVA 26) to relate to bite marks on some corollas collected opportunistically on the ground. Highly accurate measurements were not possible in view of the flexibility of the corollas, their rounded shape, their rapid degradation on the wet forest floor, the different impressions caused by the multiple layers of petals, the occasional multiple handling by one bat, and our inability in some cases to determine whether a measurement was between canines on one jaw or both jaws, so we report only on bite marks that could be measured with acceptable accuracy (i.e., representative of reality in view of the constraints).

### Presence of nectar

In 2016, after removing their corollas, we checked 14 flowers from 7 trees (1˗7 flowers per tree) once between 2240 h and 0235 h for the presence of nectar using a pipette that we adjusted until all the flower’s nectar placed in a tube fit perfectly in the tip (no air gap at the tip). In 2017, we checked 51 bagged flowers between 1820 h and 0642 h, including 36 from tree 54, and 1˗7 flowers per tree for six other trees. We tested 11 trees in total. We averaged the data for flowers from the same tree that contained a measurable quantity of nectar so that they would be represented as one flower, and here present mean (± standard deviation) nectar volumes and sugar concentrations for 2016 and 2017 flowers (n represents the number of trees with one “averaged” flower). We measured nectar sugar concentration in °Bx (g sucrose equivalent in 100 g of solution) with a temperature-compensated Reichert hand refractometer, model 10431, Leica Inc., Buffalo, U.S.A.); shadows and banding were common, so we retained the strongest reading.

### Data analysis

We determined whether any of the unvisited flowers (corollas not removed) at Waisali (n = 31) and Colo-i-Suva (n = 52 in 2016, n = 18 in 2017) produced a viable fruit by monitoring them until they fell off the tree (abortion). To calculate visitation rate, we divided natural pollination (corollas removed) by the total number of flowers available for visitation (e.g., not used for manipulations by us), including ones subsequently lost, at Colo-i-Suva in both 2016 and 2017. We compared visitation rate between the two seasons and pollination success in 2016 (summer) between bat pollination and self-pollination with two-way Chi-square tests. We considered data to be independent because we saw no evidence that fruit set may be affected by individual trees. We calculated pollination success for visited flowers in 2016 as number of ripened fruits (open) divided by the number of naturally pollinated flowers (corollas removed). To determine whether bat visitation may affect time to abortion, we compared median days to abortion between bat-visited for 2016 (n = 18) and failed flowers (not bat-visited, n = 46) with a two-sided Mann-Whitney U-test (IBM SPSS Statistics for Windows version 23.0, Amonk, U.S.A.). In view of the bias towards the observation of prompt abortions, we did not make this comparison for 2017. We grouped the cross-pollination and self-pollination fruit sets for 2016 and 2017 (assuming that developing fruits that were still present on 10 August 2017 were viable) and conducted a two-way Chi-square test to compare treatment success. Since we could not follow the fruits to their maturity in 2017, we comment on the number of closed calyxes (fruits) still present when monitoring stopped and the median number of days since pollination. Data not presented in the text appear in S2 Appendix.

## Results

### Flowers and visitors

**Fig 2:**
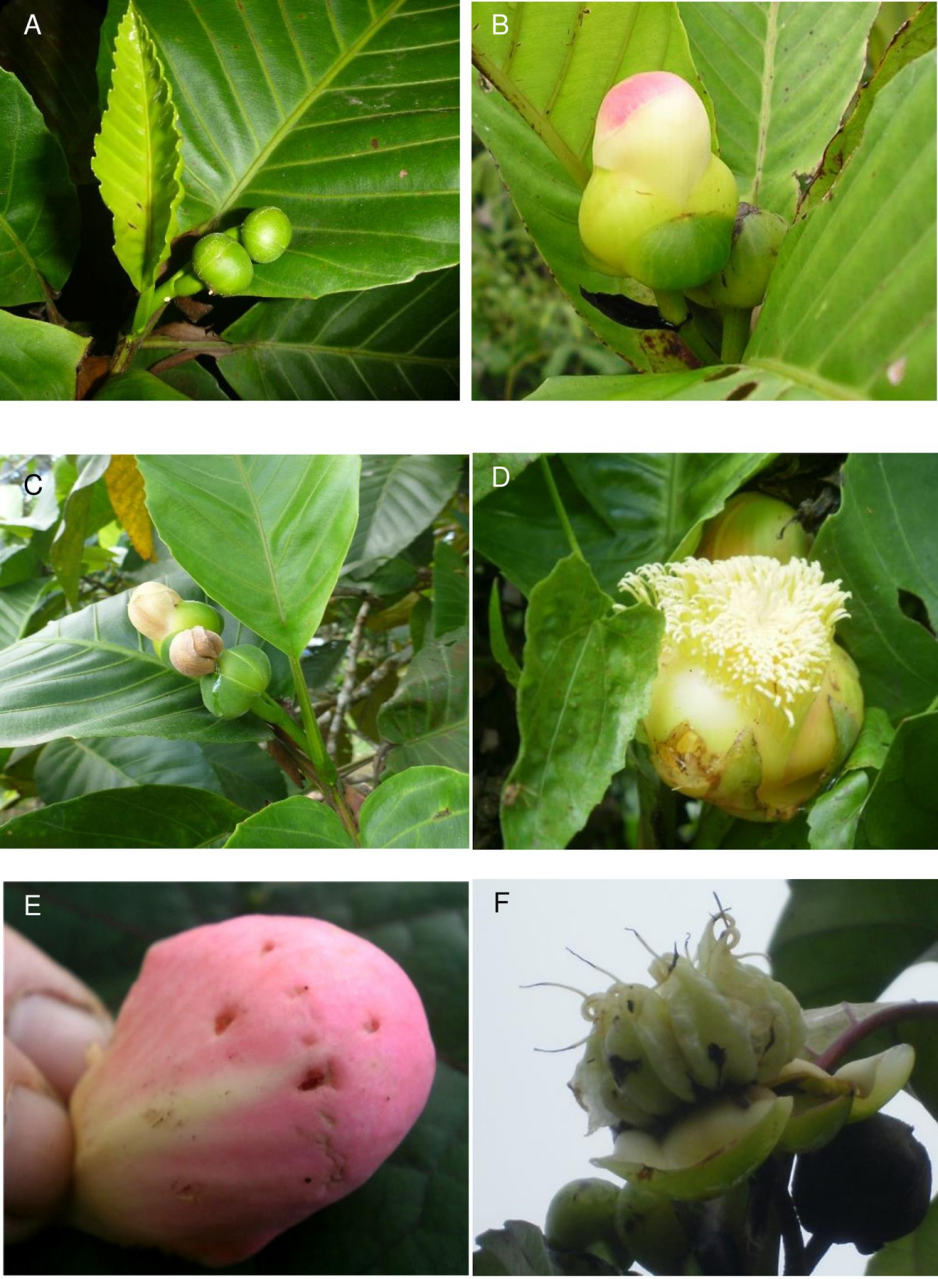
Reproductive development in *Dillenia biflora*. *(A) Pair of buds in* terminal leaf whorl. (B) Globose corolla on virgin flower (creamy white to light pink). (C) Failed flowers from no bat visit; sepals closed trapping corolla. (D) Open multi-staminate flower, corolla removed by bats. (E) Detached corolla with bat bites. (F) Mature open fruit.

As in other species of *Dillenia* [10], *D*. *biflora* flowers are large and stout, with one-day anthesis. For the flowers of *D*. *biflora* (n = 10 trees), mean (± SD) corolla height, width, and length were, respectively: 16.2 ± 3.22 mm, 17.7 ± 2.66 mm, 20.0 ± 3.48 mm. Mean calyx height, width, and length (± SD) were, respectively: 17.4 ± 1.96 mm, 26.6 ± 3.69 mm, and 28.7 ± 3.95 mm. Monitoring showed that buds started as small green spheres that enlarged over time until the yellowish cream-colored and sometimes pinkish petals appeared. The globose corollas protected the gynoandroecium completely, without any petal opening. The flowers were mature for only one night (i.e., the corolla was fully raised, available for removal, and often lightly scented). Removal of the corolla by visitors exposed the many stamens and multi-lobed stigma, and failure to remove it after the first night or following morning resulted in the death of the flowers, whose sepals constricted the base of the corollas. Fruits opened when ripe, exposing small black seeds encased in translucent white arillate flesh (Fig 2; Fig A in S1 Appendix).

We observed 8 flower visits by invasive rats (*Rattus* sp. tooth marks, scats) at Waisali. The flowers were destroyed (Fig B in S1 Appendix). In 2017, we filmed 10 corolla removals by *N*. *macdonaldi* (e.g. Fig C in S1 Appendix; [30]) and witnessed one directly (total = 11). After landing on the whorl of leaves or crawling to it, a bat bit the corolla (sometimes more than once), pulled it, and flicked it off by throwing its head back. One flower was visited 3 times while the camera was filming over 4.5 h (58 min between first two visits, and 31 min between the second and third visits). One flower received a visit 113 min after the first one. All other flowers each received a single visit when the cameras were on. For the 10 filmed flowers, cameras filmed for 4 h 14 min (± SD 19 min) on average. Corolla removal of the observed flowers took place between 1916 h and 0005 h (median = 2029 h, n = 11), but a flower we were going to film had been visited by 1845 h, indicating that bats were active immediately after dark (sunset ~ 1740 h; end of astronomical twilight ~ 1858 h) and were residing in the vicinity. Five flowers were visited before we positioned a camera (1845 h to 2220 h) and 16 after we removed the cameras, including after 0400 h. Time of discovery can be late into the night, and it is also possible that cameras may have disturbed the bats, although filmed bats showed no sign of them being disturbed. The median approach time to a flower (first visit) was 16.5 s (IQR = 7.00), the median corolla handling time was 8.0 s (IQR = 3.75), and the median feeding time was 37.0 s (IQR 14.00), with a range of 18˗84 s, representing generally more time for pollination than our hand pollination of ~ 20 s. None of the bat-visited flowers was damaged. Bats spent less time feeding during the second and third visits at the two flowers that had repeated visits.

We did not see *P*. *samoensis* at the Forest Park during our study. *Pteropus tonganus* flew over the Forest Park every day, as early as 1500 h, but we did not observe them feeding on *D*. *biflora*, in the mid-stratum of the forest. We observed individuals feeding on different trees in more open areas, just outside of the park. Moths and geckoes were very common visitors. Moths (including those monitored on 17 flowers) were not capable of removing the corollas, and none of the monitored geckoes, which visited 23 closed flowers, removed the corollas, including large oceanic geckoes (*Gehyra oceanica*). Moths occasionally seemed to insert their proboscis along the corollas to reach the nectar, and geckoes sometimes licked the sides of the flowers at the joint between corolla and calyx. When the corollas were removed, moths and geckoes fed on nectar while positioned on the side of the flowers, without brushing against the anthers or stigma, and one gecko appeared to be eating anthers from its position on the side of the flower. We recorded an instance of an altercation between a gecko “guarding” a flower (head on flower) and a bat that screeched and excluded the gecko with head and wing movements before removing the corolla (Fig C in S1 Appendix; [30]). The gecko remained in the vicinity of the flower.

We frequently found discarded corollas on the ground; they generally were pierced or bruised where the four canines of a bat had grabbed them (Fig 2; Fig D in S1 Appendix). The *N*. *macdonaldi* specimens lodged at the University of the South Pacific, like the 193 trapped live by Scanlon and Petit [26], had greatly developed canines (Fig E in S1 Appendix), but reduced cheek teeth suitable for bats that do not consume fruits and specialize on floral nectar and pollen [13, 24, 31]. The measurements between canines of 4.00 mm (top and bottom, juvenile male specimen), 5.90 (top) and 5.62 (bottom), and 15.12 mm between the top and bottom canines (adult female specimen) matched the bite marks that we observed on curved corollas (n ≥ 14; measurements varied between 4.8 and 10.8 mm).

### Pollination

From 10 trees at Waisali and 20 at Colo-i-Suva, all 101 *D*. *biflora* flowers (Vanua Levu n = 31, and Colo-i-Suva n = 70, Table 1 and Table A in S1 Appendix) that had kept their corolla aborted, demonstrating that this species is not cleistogamous. All pollination (represented by the removal of corollas) was nocturnal, and the rate of corolla removal was 28/84 (33%) in 2016 (summer), and 46/73 (63%) in 2017 (winter) (χ^2^_1_ = 15.765, p < 0.01). We found no evidence of difference in outcome between self-pollination and cross pollination for 2016 and 2017 combined, assuming that fruit presence on 10 August 2017 was representative of outcome (χ^2^_1_ = 0.005, p < 0.05). In 2016, ripe fruits (dehisced) had a median age of 38 d (IQR = 4; n = 20). Just 20% (n = 25) of flowers with corolla removed by bats set fruit, as did 83% (n = 12) of the flowers we self-pollinated by hand nocturnally (χ^2^_1_ = 13.492, p < 0.01), the highest fruit set of all treatments (Table 1). Two of 5 flowers (40%) that we self-pollinated at dawn (0555 h to 0610 h) set fruit (Table 1). Fifty-seven percent of the seven flowers we cross-pollinated at night set fruit. Abortions of bat-visited flowers (median = 6.5 d, range = 3˗26 d) took place later than those of flowers that had not been visited (2016 median = 4.0 d, range = 1˗15 d; U = 237.5, N = 64, p = 0.006; Fig F in S1 Appendix.). Although we could not follow the fruits to maturity in 2017, three of four cross-pollinated fruits were still alive when we left the site, and 18 of 31 self-pollinated fruits (Table A in S1 Appendix). In 2017, only 17% of the fruits resulting from bat visitation were still present when we left the site (n = 42; 27˗41 d) and 58% of the fruits resulting from self-pollination by hand (n = 31, 29˗43 d and one collected open at 47 d; 7 fruits > 40 d were still on the trees, indicating that ripening may have been delayed in winter). Three of four cross-pollinations by hand had formed fruits that were still there (35˗43 d).

**Table 1:** Pollination treatments and percentage fruit set for *Dillenia biflora* flowers in 2016.

Treatments summer 2016	number of flowers	fruit	no fruit	% fruit set
Natural pollination (corolla removed at night)	25	5	20	20.0
Flowers that kept their corolla (unvisited)	50	0	50	0
Flower with corolla removed by us at night and bagged*	1	0	1	0
Flower with corolla removed by us during the day*	2	0	2	0
Flower bagged 24 h*	2	0	2	0
Cross-pollination by hand at night (with or without nectar collection)	7	4	3	57.1
Self-pollination by hand at night (with or without nectar collection)	12	10	2	83.3
Self-pollination by hand in the morning (with or without nectar collection)	5	2	3	40.0
Flowers from which we collected nectar at night	9	2	7	22.2
Flowers from which we collected nectar in the morning	6	0	6	0

*In view of the paucity of accessible flowers, these treatments were designated low priority since we promptly established that pollination required corolla removal and interference with the stigma.

### Nectar presence in *Dillenia*

In 2016, all 14 bagged flowers contained nectar. The maximum was 1140 μL (0000 h); the mean was 483.0 ± 296.15 μL (n = 6, including averaged data for the same trees). Nectar sugar concentration was 9.3 ± 2.68%. In 2017, three out of 51 bagged flowers from seven trees had no nectar, but came from trees that had other flowers with nectar. Two had small drops that were not measured. The maximum amount was 2190 μL (one collection at 2020 h) and the mean was 650.7 ± 227.34 μL (n = 5, including averaged data for the same trees). Sugar concentration was 10.7 ± 0.70%.

## Discussion

Remarkably, *D*. *biflora* flowers bear corollas that never open. They are not cleistogamous, but are visited by bats that remove the corollas. Bats are obligate visitors of *D*. *biflora* for pollination. At our sites on two Fijian islands, every single one of 101 flowers that retained its corolla died, and only bats removed the corollas without destroying flowers, based on 224 h of filming, direct observation, and tooth marks on corollas.

We have no evidence that cross-pollination and self-pollination by hand differed overall. It is likely that self-pollination by hand was more successful than was pollination by bats because flowers were bagged after self-pollination, whereas bat-visited flowers were not, leaving increased access to parasitic insects after corolla removal. Additional evidence for a possible involvement of parasitic insects in abortions includes the fact that abortion rates were significantly delayed for bat-visited flowers compared to unvisited flowers, indicating that a mechanism other than inadequate pollen delivery may be a consideration. Insufficient pollen load is unlikely to be an explanation, considering the time spent by bats at flowers and lack of damage to flowers. Numerous aborted fruits that we found on the forest floor showed insect damage. Insect parasitism is prevalent in Malayan Borneo where 53% of *D*. *suffruticosa* fruit abortions were due to insects [32]. The impact of such parasitism in rainforests may be underestimated and could be tested by bagging bat-visited flowers immediately after visitation. Rain in the open flower could also affect pollination and fruit development, particularly in the wet season (e.g., effect of rain [33] and references therein). Regardless, we demonstrated that fruit formation was possible by mediated autogamy after corolla removal; the fruits had fully formed seeds similar to those resulting from natural pollination.

The greater bat visitation to *D*. *biflora* flowers that we observed in 2017 (winter) may reflect the paucity of other resources that are available in winter or the fact that we marked the flowers after they had matured, rather than before. This effort to minimize bat disturbance may have had a positive effect on visitation if some bat individuals responded negatively to the presence of the tags. Scanlon et al. [34] showed that bat resources were less abundant in secondary forest in winter than in summer on Vanua Levu, suggesting that low bat resource availability may increase the attractiveness of flowers in winter.

The only visitors capable of facilitating pollination were bats. Geckos and moths did not remove the corollas; it is possible that other taxa than bats may assist pollination (e.g., birds and bees in early morning), but bats are necessary to open the flowers. We do not know whether *P*. *tonganus* (commonly seen flying above the forest) and *P*. *samoensis* feed in the same way as does *N*. *macdonaldi* and affect the integrity of *D*. *biflora* flowers, but these flowers are not of primary interest to them [13], possibly because flowers tend to occur in cluttered environments, within the forest’s canopy. We suggest that further studies should examine the relationships of different bat species with *D*. *biflora*. The only bat that we recorded visiting the flowers at Colo-i-Suva was *N*. *macdonaldi*. A cave containing *N*. *macdonaldi* exists in the village of Kalabu (Naitasiri Province), adjacent to Colo-i-Suva Forest Park, possibly explaining the early arrival on site of the bats. Cave proximity can affect tree pollination [35] and it would be interesting to determine how the presence of bat roosts affects *D*. *biflora* pollination throughout its range. It is noteworthy that *N*. *macdonaldi* is also sympatric with *D*. *biflora* in Vanuatu, but was extirpated by humans from ‘Eua, Tonga, where bones were found in a cave stratum dated ~ 570˗2700 yr before present [36], and it is not known whether the plant ever existed in Tonga. The *D*. *biflora* corollas that we found on the forest floor in Fiji bore marks that matched this bat’s dentition. *Notopteris macdonaldi*’s dentition seems to serve a relatively strong specialization on *D*. *biflora* flowers, although it also feeds extensively on *Alpinia* spp. (Zingiberaceae), *Barringtonia seaturae* (Lecythidaceae), and *Syzygium* spp. (Myrtaceae) [13], which have different flowers structures. Some species in the Lecythidaceae have flowers reminiscent of those of *D*. *biflora* and are pollinated by bats [37], but corollas appear to open unaided. *Melonycteris*, which does not occur in Fiji, has similar canines and cheek-teeth to those of *Notopteris* [25] and occurs in the range of other *Dillenia* species, where it could facilitate pollination of some of those species (see below).

A key characteristic of Dilleniaceae was the absence of nectary [7, 8]. However, we found abundant nectar in almost all flowers we sampled once only (means were 483 μL in 2016 and 651 μL in 2017), representing large volumes expected from bat flowers [38–40]. *Dillenia biflora* clearly produces copious amounts of nectar as a reward for bats, which must visit the flowers before pollination can take place. Since the presence of nectaries was not known for the family, this character has not been used in the elucidation of phylogenetic relationships, which remain unresolved for Dilleniaceae [8]. At around 10%, sugar concentration in *D*. *biflora* was low relative to some species visited by flying-foxes (e.g. *Bombax ceiba*, 19˗25% [41]; *Ceiba pentandra*, 10˗26% [40])), but was in the range of others (e.g. *Corymba gummifera*, 9.3% [42], three *Durio* species, 5˗16% [43]). We collected nectar at different times of night, and it is possible that nectar may be more concentrated and abundant in the first part of the night, as is the case in other species [e.g., 39, 43].

It is likely that nectar production and bat pollination are not traits exclusive to *D*. *biflora* within the Dilleniaceae. In his treatment of the genus, Hoogland [11] noted for this species “*flowers probably never quite expanding*” and “*petals falling off without spreading*,” which must refer to corollas found on the ground after removal by bats. Inspection of the paper and additional notes [44] reveals other species with this description: *D*. *schlechteri* (E New Guinea and New Ireland), *D*. *papuana* (New Guinea and islands in the region), *D*. *quercifolia* (SE New Guinea), *D*. *montana* (E New Guinea), *D*. *nalagi* (Papua, reportedly very similar to *D*. *biflora*), *D*. *cyclopensis* (Papua), *D*. *crenata*, *D*. *salomonensis*, and *D*. *insignis* (Solomon Islands), and *D*. *pteropoda* (Philippines, Moluccas). In addition, other flowers may deserve further scrutiny, such as those “incompletely known” (*D*. *ingens*, Solomon Islands), “*known only from buds*” (*D*. *fagifolia*, New Guinea), and “*petals unknown*” (*D*. *marsupialis*, Philippines; *D*. *insularum*, E New Guinea islands). Hoogland [11] wrote “*The remarkable corolla which falls off as a whole without spreading is distributed throughout the easternmost part of the area* [range of *Dillenia*] *(mainly in and E of New Guinea)* [comment revised in Hoogland [44] to include species further west]*; the fully expanding corolla is perhaps not found E of New Guinea*, *but not all species from this area are sufficiently known to indicate exactly the eastern limit of the occurrence of this character*.” Numerous species of Pteropodidae occur in the above localities, some of which could be suitable pollinators of *Dillenia* spp. In any case, *D*. *biflora* occurs only in Fiji and Vanuatu, the only places where *N*. *macdonaldi* also occurs. It is likely that several or even many *Dillenia* species, believed to be nectarless and bee-pollinated [7], are actually chiropterophilous or chiropteropisunous, from chiropteropistusis (“relying on bats”) for plants with corollas that must be removed by bats. It is an exciting discovery for the understanding of angiosperm evolution and many *Dillenia* species should be studied at night in an effort to understand their mode of pollination. Petal micromorphology may also help to identify pollinators of this genus by providing clues regarding chiropterophilous attributes [45].

The presence of nectaries in *Dillenia* and chiropteropisteusis may provide new clues for evolutionary biologists working on the evolution of Dilleniaceae and an improved understanding of the ecological and conservation links between bats and forests. *Notopteris macdonaldi* may not be the only one that has a close association with the genus *Dillenia*. For example, *Macroglossus*, *Eonycteris*, *Melonycteris*, and *Syconycteris* could also enlighten the evolution of *Dillenia* and the conservation of ecosystems in which it is extant if chiropteropisteusis occurs in other species of this genus, as we predict from Hoogland’s notes [11, 44]. Several species in those bat genera have decreasing populations according to the IUCN Red List (accessed 28 December 2020). The extinction of bats would lead to the extinction of chiropteropisunous plants (see also plant widowhood [46]). Similarly, the extinction of *D*. *biflora*, represented in 70% of *N*. *macdonaldi* dietary samples [13] would certainly have a large impact on the population of this blossom bat, already affected by other threats. Three *Dillenia* species with corollas “falling off without spreading” (*D*. *schlechteri*, *D*. *papuana*, *D*. *salomonensis*) are among the assessed *Dillenia* species that are threatened or have declining populations (17 of 22 species on the IUCN Red List accessed 28 December 20).

Many unusual cases of pollination by bats exist, such as the pollination of *Gongylolepis martiana* (Asteraceae, a family mostly pollinated by invertebrates) [47] and members of the Acanthaceae [48]; however, they involve classic anthesis − i.e., flowers opening when sexually functional, as is the case for most known bat-pollinated flowers [37]. Two other examples of pollination requiring corolla manipulation by bats include the explosive pollen release of *Mucuna holtonii* [49] and *M*. *andreana* [50] by phyllostomid bats in Costa Rica, and corolla opening of *Mucuna* species by flying-foxes [51, 52]. In the case of *M*. *holtonii* and *M*. *andreana*, the bat grasps the flower and inserts its snout into a slit in the corolla for the flower to release pollen explosively. In contrast, the hard petals of other *Mucuna* species are opened explosively by the pressure of a flying-fox’s snout at the base of the petals. For example, *M*. *macrocarpa* is opened by Orii’s flying-fox *Pteropus dasymallus inopinatus* [53], but also different mammal species according to region [51 and references therein]. *Mucuna championii* is pollinated by two mammal species including a flying-fox within one location [52]. Other species may be pollinated exclusively by bats, such as possibly *M*. *macropoda* in Papua New Guinea [54]. A plant should be considered chiropteropisunous only when bats are entirely responsible for the manipulation of flowers in such a way that pollination is mediated.

Chiropteropisteusis appears to be unique among the pollination systems involving flower manipulation. Examples of pollination facilitated by vertebrate manipulation exist, but flowers do not depend entirely on the visitors. Visitation by *Eonycteris spelaea* sped up the opening of *Oxylum indicum* in Malaysia, and the range of both species overlapped [55]. The anther-bearing corolla of small honeypot flowers (*Acrotriche* spp., Ericaceae) in Australia can be removed by western pygmy-possums (*Cercartetus concinnus*) that may facilitate pollination by exposing the stigma [56], because most of the nectar reward is associated with the corolla [57], but the corolla does not necessarily need a visitor for removal [58]. The *Peraxilla* mistletoes of New Zealand also benefit from animal manipulation (*P*. *tetrapetala* and *P*. *colensoi*, Loranthaceae) [59, 60]. Native honeyeaters grasp the tip of ripe buds and twist, triggering an explosive opening of the flowers, which can self-pollinate if unopened [59, 60]. Other mistletoe (*Loranthus*) flowers that seemed to require bird manipulation for pollination were recorded in Africa (sunbirds split open closed corolla tubes triggering an explosive opening [61]) and western India (sunbirds squeezed the tops of mature buds causing them to fling open [62]).

## Conclusions

Unusual flower structures improve pollinator constancy [5]. *Dillenia biflora* flowers with closed corollas can wait for the best (and possibly only worthwhile) pollinators, while protecting pollen and nectar from the rain, and the flowers from parasites. Considering the alarming threats to island bats [14, 63] and the reliance of bat plants on their pollinators [64], bat conservation is crucial, particularly for chiropteropisunous plants. Such plants may also make a significant contribution to bats’ diets and threats to them (e.g., mining, logging, and clearing to *D*. *salomonensis* [65] if it is chiropteropisunous), could affect dramatically local bat populations. When we conducted this study, the Ministry of Forestry in Fiji intended to conduct selective logging of *D*. *biflora*, but was receptive to our findings. Future work is likely to identify several more *Dillenia* species that depend entirely on bats, and open new avenues of research for managing tropical agroforestry, protecting natural systems, and for the understanding of the evolution of angiosperms. Long-ignored nocturnal botany still holds many secrets.

## Supporting information

S1 Appendix Supplementary in-text information: Text A in S1 Appendix. **Weather information for the Viti Levu study area. Fig A in S1 Appendix. Ripe *Dillenia biflora* fruit. Fig B in S1 Appendix. *Dillenia biflora* flowers visited by rats.** Exposed androgynoecium with sepals masticated by rats (left), and the removed corolla and damaged sepals of another rat-destroyed flower showing rat scats and chewed flower parts (right). **Fig C in S1 Appendix. Pollination of a *Dillenia biflora* flower by *Notopteris macdonaldi*.** (A) Approaching bat. (B) Bat fights with gecko after landing. (C) Gecko moves away and bat positions itself to remove the corolla. (D) Bat grabs the corolla in its mouth. (E) Bat pulls the corolla off. (F) Bat flicks its head and body back (ventral side visible) to throw out the corolla. (G) Bat feeds on the flower. **Fig D in S1 Appendix.** *Dillenia biflora* petals found on the forest floor and bearing *Notopteris macdonaldi* tooth marks. **Fig E in S1 Appendix. Specimen AXM 001 (University of the South Pacific) adult female *Notopteris macdonaldi*, showing long canines and reduced other teeth. Fig F in S1 Appendix.** Days to abortion for bat-visited flowers (n = 18) that did not produce fruits and for flowers that were not visited (n = 46) (2016; × indicates mean). **Table A in S1 Appendix. *Dillenia biflora* pollination treatments and fruits set (n = 1) or persisting at 10 August 2017.**
(PDF)

S2 Appendix Data for flower measurements, filmed bat visits, days to ripe fruits 2016, and flower abortion days post-pollination. (XLSX)
