# Supplementary material for: A novel bat pollination system involving obligate flower corolla removal has implications for global Dillenia conservation
Source: PLoS One. 2022 Feb 3;17(2):e0262985. doi: 10.1371/journal.pone.0262985 (PMC8812880; doi:10.1371/journal.pone.0262985)
Supplement: S1 Appendix — Weather information for the Viti Levu study area. Fig A in S1 Appendix. Ripe Dillenia biflora fruit. Fig B in S1 Appendix. Dillenia biflora flowers visited by rats. Exposed androgynoecium with sepals masticated by rats (left), and the removed corolla and damaged sepals of another rat-destroyed flower showing rat scats and chewed flower parts (right). Fig C in S1 Appendix. Pollination of a Dillenia biflora flower by Notopteris macdonaldi. (A) Approaching bat. (B) Bat fights with gecko after landing. (C) Gecko moves away and bat positions itself to remove the corolla. (D) Bat grabs the corolla in its mouth. (E) Bat pulls the corolla off. (F) Bat flicks its head and body back (ventral side visible) to throw out the corolla. (G) Bat feeds on the flower. Fig D in S1 Appendix. Dillenia biflora petals found on the forest floor and bearing Notopteris macdonaldi tooth marks. Fig E in S1 Appendix. Specimen AXM 001 (University of the South Pacific) adult female Notopteris macdonaldi, showing long canines and reduced other teeth. Fig F in S1 Appendix. Days to abortion for bat-visited flowers (n = 18) that did not produce fruits and for flowers that were not visited (n = 46) (2016; × indicates mean). Table A in S1 Appendix. Dillenia biflora pollination treatments and fruits set (n = 1) or persisting at 10 August 2017. (PDF) [file pone.0262985.s001.pdf]

SUPPORTING INFORMATION: S1 APPENDIX

A novel bat pollination system involving obligate flower corolla removal has implications for global *Dillenia* conservation

Sophie Petit, Annette T. Scanlon, Alivereti Naikatini, Tara Pukala, Russell Schumann

S1 Appendix. Supplementary in-text information.

Text A in S1 Appendix. Weather information for the Viti Levu study area.

Reported mean daily temperature ranges were 23- 29°C in January and 19−25°C in July for Colo-i-Suva, with relative humidity in the forest at dawn ~90-100% and mean annual rainfall of 4100 mm [1,2]. Rainfall at Laucala Bay (~ 9 km from Colo-i-Suva) in November (118.0 mm) and December 2016 (761.8 mm) represented 48% and 275% of the normal average (1971-2000), respectively [3]. At Laucala Bay, 110 mm of rain fell in June 2017[4] and 85 mm in July[5]. Average maximum temperatures were 30.4°C in November, 30.0°C in December 2016, 27.5 °C in June, and 26.5°C in July 2017 [3,4,5].

1. Ash J. Demography and production of *Leptopteris wilkesiana* (Osmundaceae), a tropical tree-fern from Fiji. 1986; Aust J Bot 34: 207−215.
2. Ash J. Demography of *Cyathea hornei* (Cyatheaceae), a tropical tree-fern in Fiji. 1987; Aust J Bot 35: 331−342.
3. Fiji Meteorological Service. Fiji annual climate summary 2016. 2017a. [Cited 2020 March 28]. Available from: http://www.met.gov.fj/Summary2.pdf
4. Fiji Meteorological Service. Fiji climate summary June 2017. (Fiji Meteorological Service, Nadi, Vol. 38, Issue 6). 2017b. [Cited 2020 March 28]. Available from: http://www.met.gov.fj/aifs_prods/FSCJUN.pdf
5. Fiji Meteorological Service. Fiji climate summary July 2017. (Fiji Meteorological Service, Nadi, Vol. 38, Issue 7). 2017c. [Cited 2020 March 28]. Available from: http://www.met.gov.fj/aifs_prods/FSCJULY.pdf

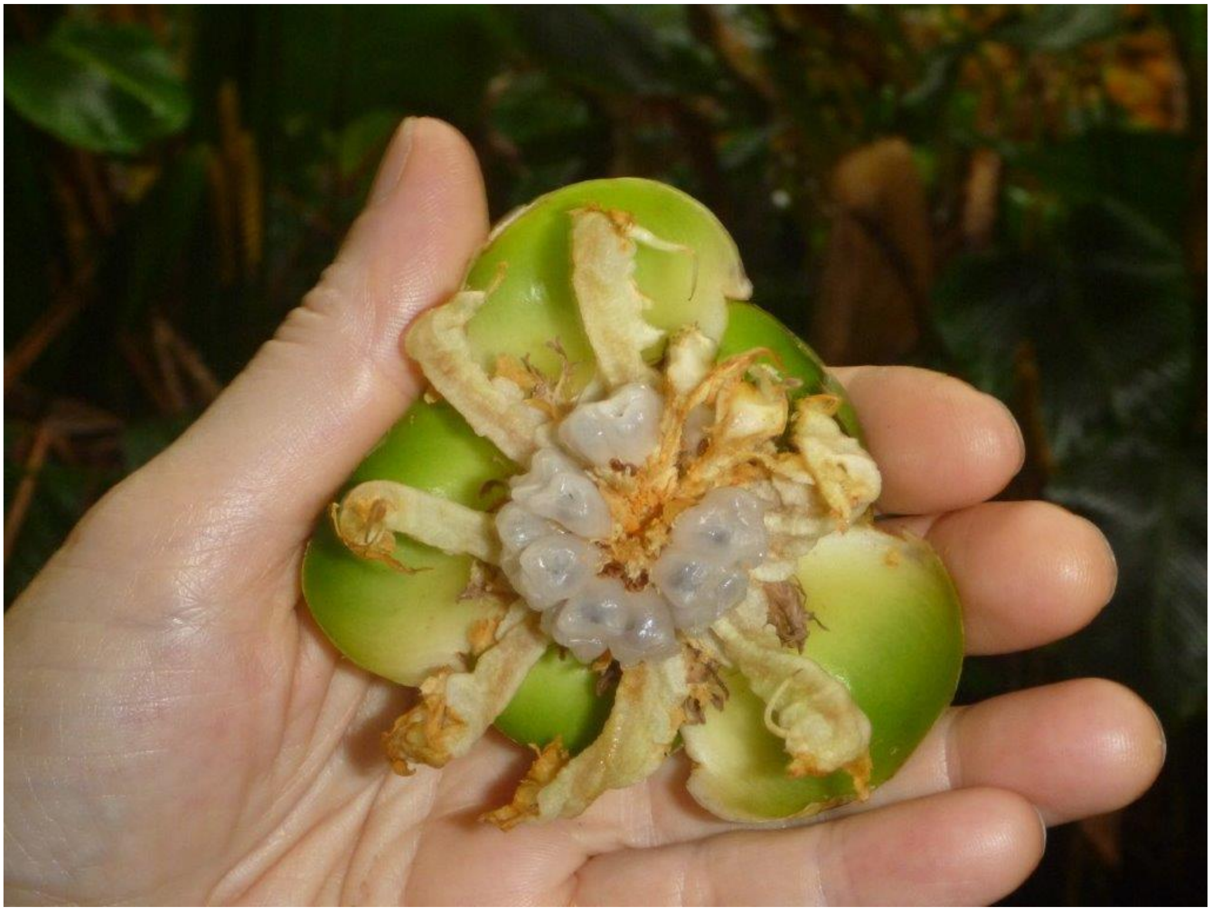

Fig A in S1 Appendix. Ripe *Dillenia biflora* fruit.

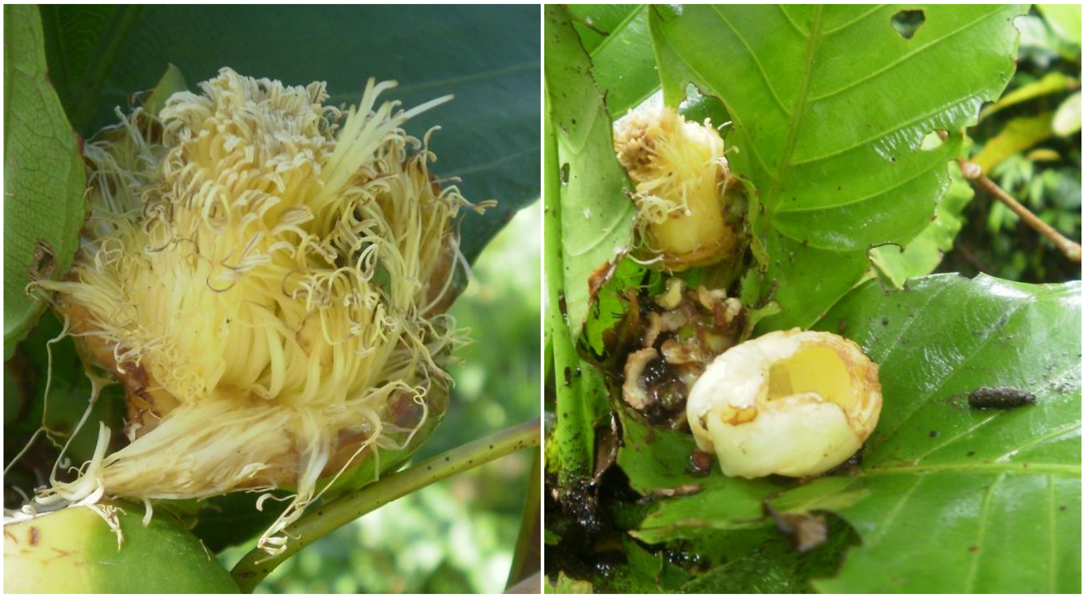

Fig B in S1 Appendix. *Dillenia biflora* flowers visited by rats. Exposed androgynoecium with sepals masticated by rats (left), and the removed corolla and damaged sepals of another rat-destroyed flower showing rat scats and chewed flower parts (right).

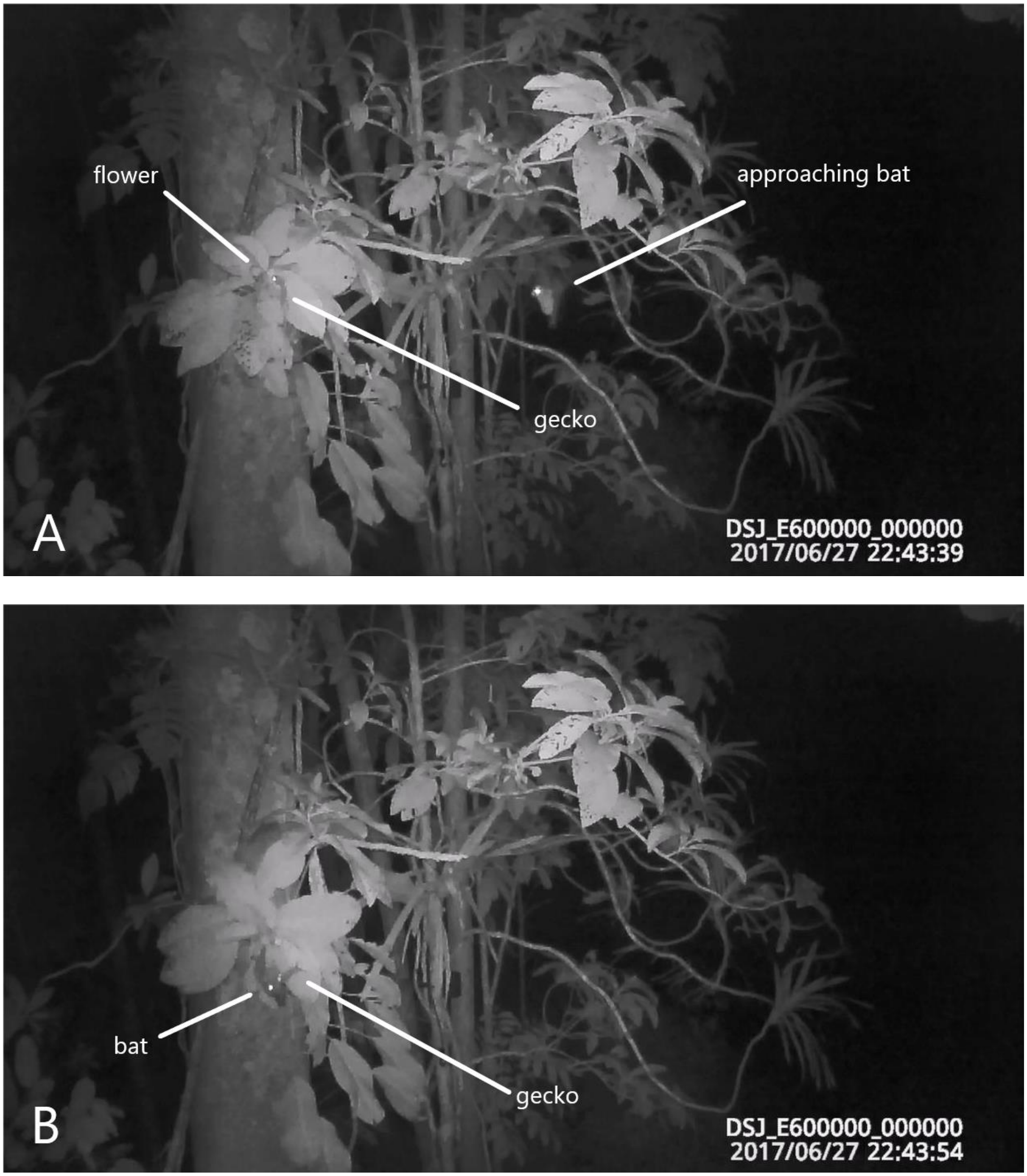
flower
approaching bat
gecko
A
DSJ_E600000_000000
2017/06/27 22:43:39
bat
gecko
B
DSJ_E600000_000000
2017/06/27 22:43:54

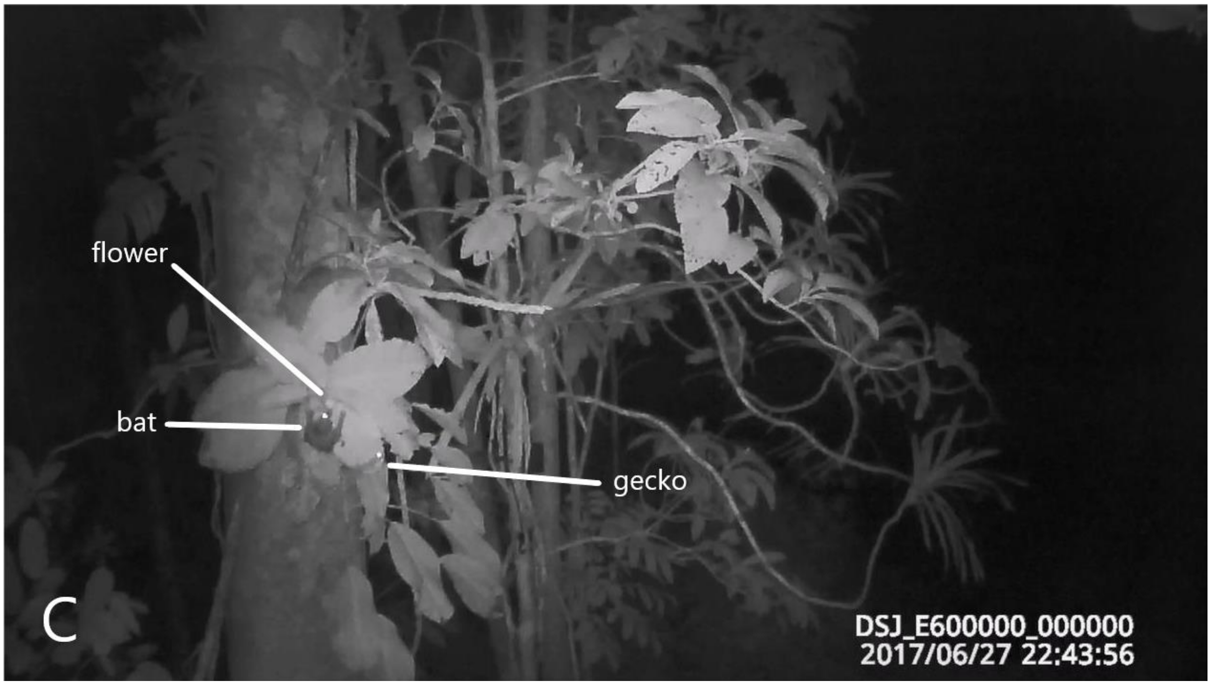
flower
bat
gecko
C
DSJ_E600000_000000
2017/06/27 22:43:56

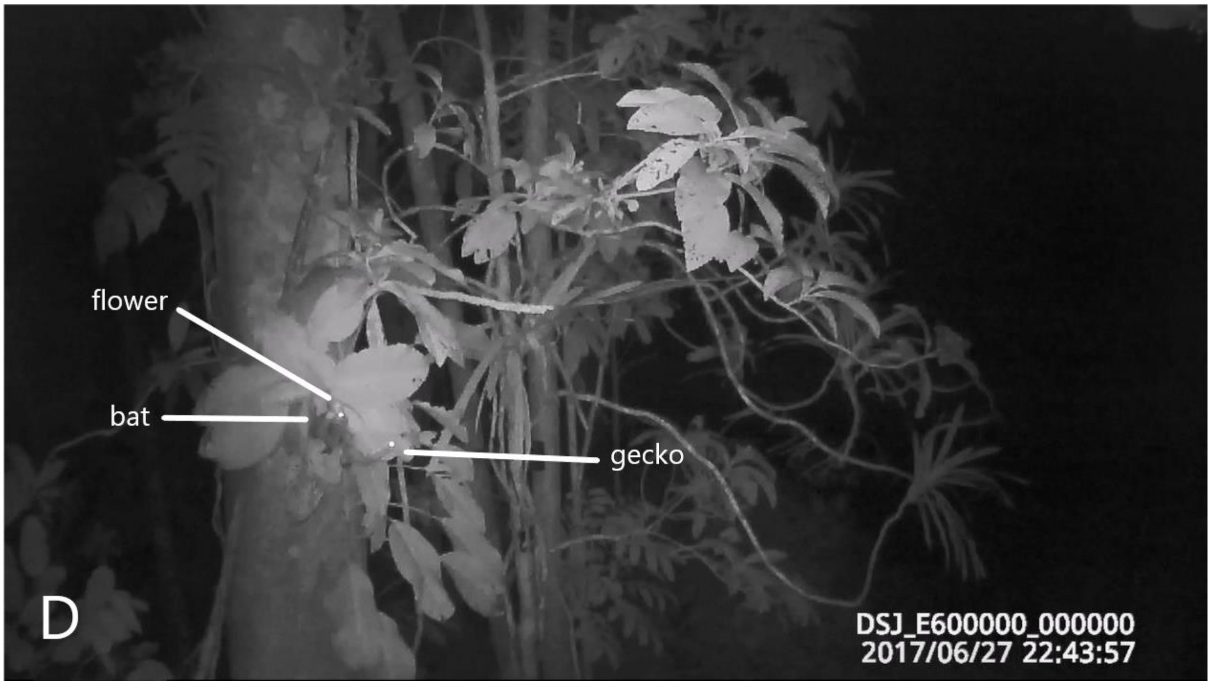
flower
bat
gecko
D
DSJ_E600000_000000
2017/06/27 22:43:57

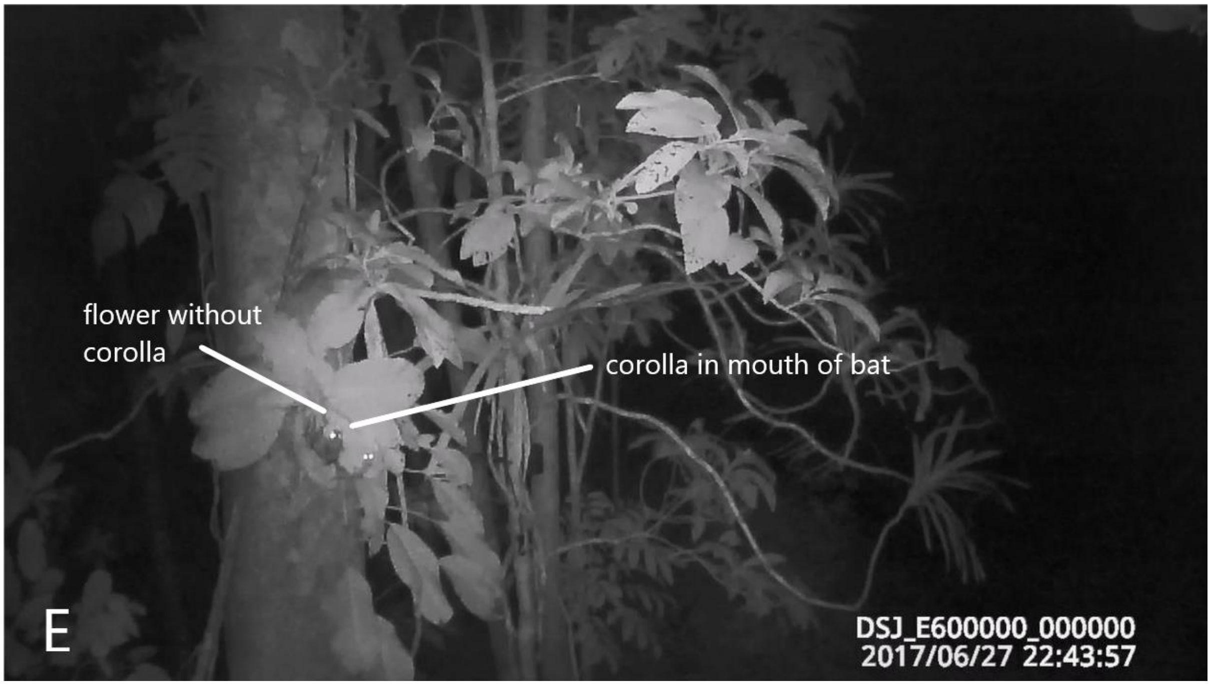
flower without corolla
corolla in mouth of bat
E
DSJ_E600000_000000
2017/06/27 22:43:57

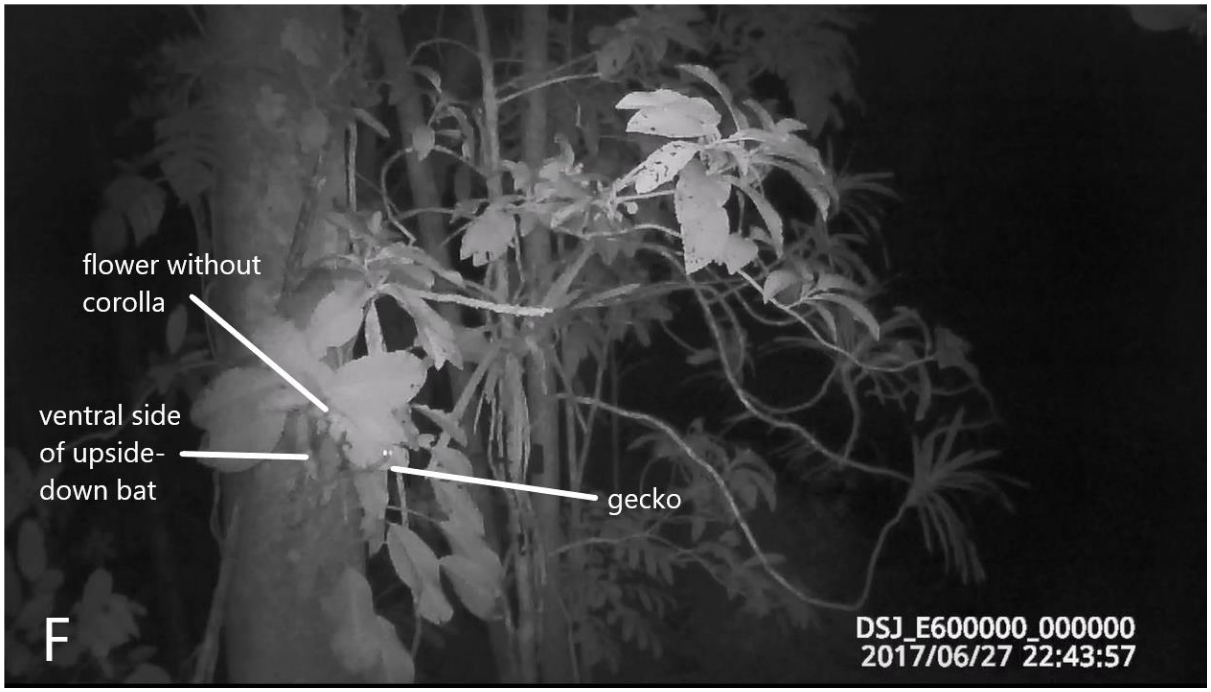
flower without corolla
ventral side of upside-down bat
gecko
F
DSJ_E600000_000000
2017/06/27 22:43:57

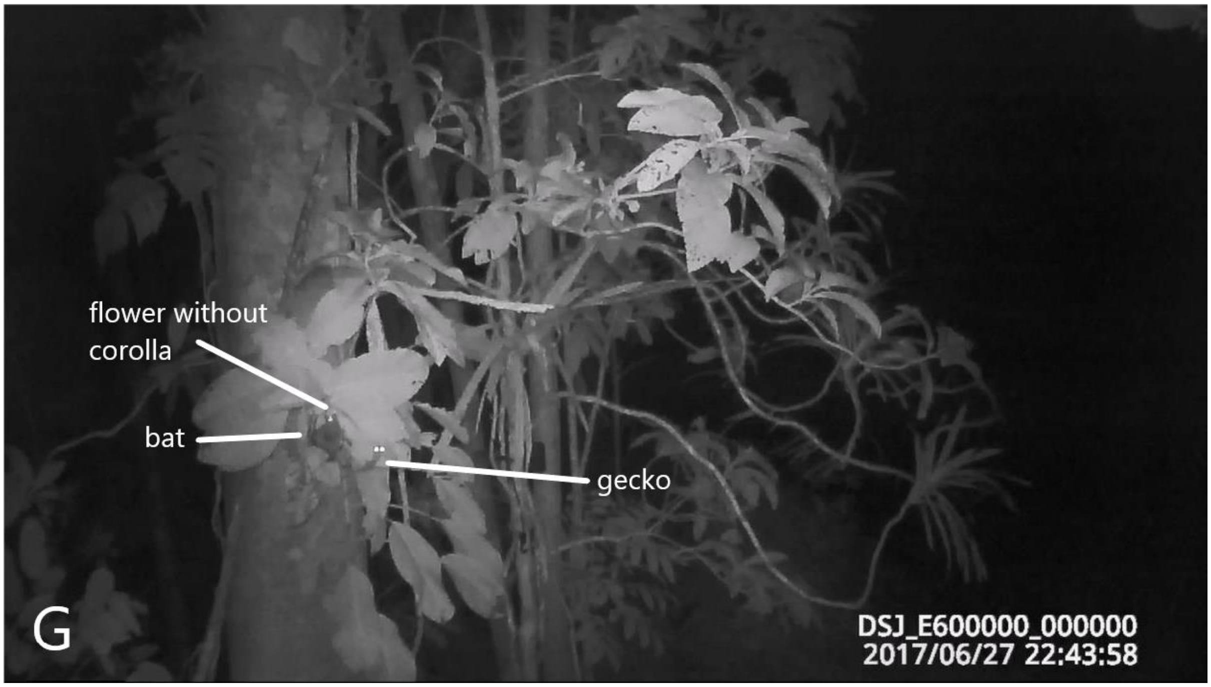

Fig C in S1 Appendix. Pollination of a *Dillenia biflora* flower by *Notopteris macdonaldi*. **(**A) Approaching bat. (B) Bat fights with gecko after landing. (C) Gecko moves away and bat positions itself to remove the corolla. (D) Bat grabs the corolla in its mouth. (E) Bat pulls the corolla off. (F) Bat flicks its head and body back (ventral side visible) to throw out the corolla. (G) Bat feeds on the flower.

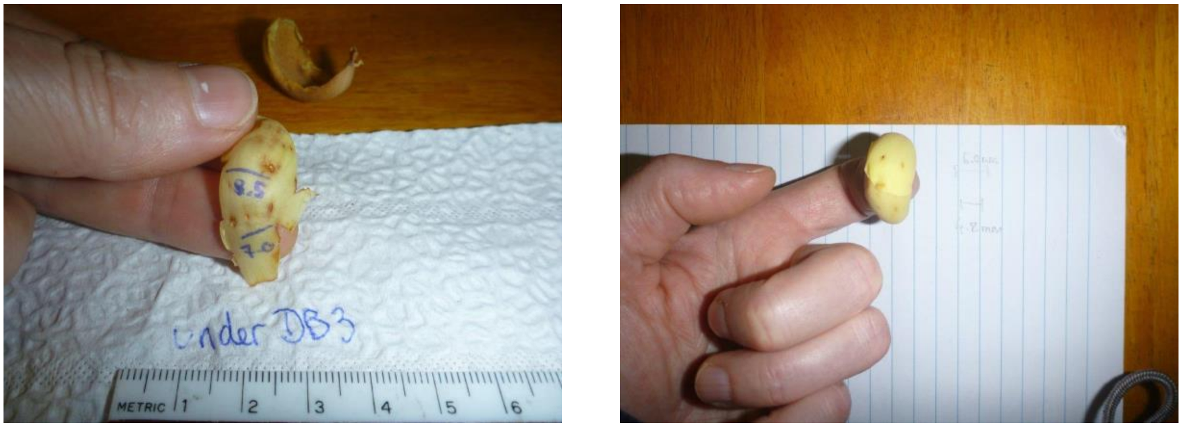

Fig D in S1 Appendix. ***Dillenia biflora*** **petals found on the forest floor and bearing *Notopteris macdonaldi* tooth marks.**

9

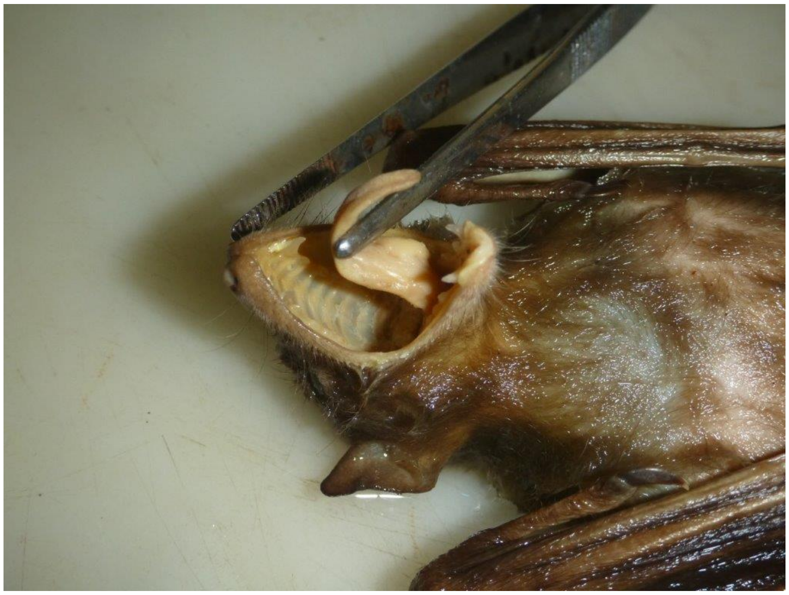

Fig E in S1 Appendix. Specimen AXM 001 (University of the South Pacific) adult female *Notopteris macdonaldi*, showing long canines and reduced other teeth.

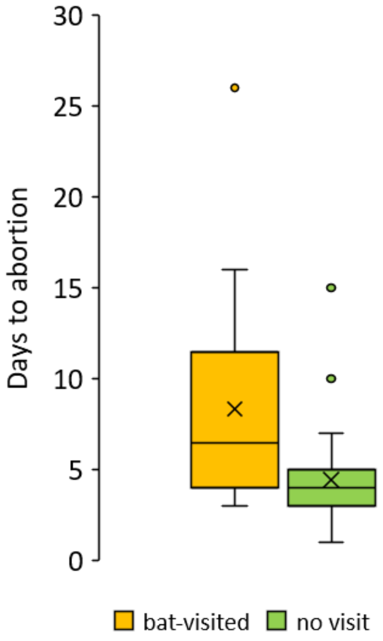

Fig F in S1 Appendix. Days to abortion for bat-visited flowers (n = 18) that did not produce fruits and for flowers that were not visited (n = 46) (2016; × indicates mean).

Table A in S1 Appendix. ***Dillenia biflora*** **pollination treatments and fruits set (n = 1) or persisting at 10 August 2017.**

**Treatments winter 2017**	number of flowers	fruit still there (or collected)	gone/aborted	% fruits still alive at 10 August
Natural pollination (corolla removed at night)	42	7	35	16.7
Flowers that kept their corolla (unvisited)	18	0	18	0
Cross-pollination by hand at night	4	3	1	75.0
Self-pollination by hand at night	31	17 (1)	13	58.1
Flowers from which we collected nectar all night	8	0	8	0
Flower from which we collected nectar once at night	1	1	0	100.0
